# Moving Education Online During the COVID-19 Pandemic: Thinking Back and Looking Ahead

**DOI:** 10.3389/fpubh.2021.751685

**Published:** 2021-10-25

**Authors:** Sergio A. Costa, Ilias Kavouras, Nevin Cohen, Terry T. K. Huang

**Affiliations:** Graduate School of Public Health and Health Policy, City University of New York, New York, NY, United States

**Keywords:** online learning, post-pandemic education, innovation in education, public health, remote learning

In the United States, enrollment in online education has increased over the last decade. By fall of 2018, one third of almost 19.7 million students enrolled in degree-granting post-secondary institutions had enrolled in online courses with nearly 40% of graduate students taking online courses (1, 2). Among 120 CEPH-accredited schools of public health, 57 schools have fully online programs and 48 have hybrid online and on-campus models (3).

During the COVID-19 pandemic, universities and colleges across the United States were forced to shift rapidly from in-person education to “emergency remote learning” with little time to redesign courses and programs appropriately (4). Several challenges made the transition difficult: limited resources due to pandemic-related costs and revenue losses; equipment and workspace constraints for students; and misunderstandings about the pedagogical differences between instructional modes. Schools and programs restricted by hiring freezes and reduced budgets made it difficult for instructors to plan, build and manage courses, and to get extra help to use new technologies. The shift to remote learning also exposed that many students lacked resources and capacity to continue their education online, including unreliable internet access and computer hardware and inadequate space and time conducive for study. Without training in online education or resources to develop different types of courses, many faculty simply tried to replicate online the methods of teaching they had been doing in-person.

The pandemic-induced shift online presents substantial problems and pressure for academic services and support units. We propose three ways that schools and programs can respond: (1) training faculty on pedagogy and the technology that enables online learning; (2) improving student readiness and preparation for online education; and (3) reimagining educational offerings that respond to skills in demand. Together, these strategies will help programs and schools keep pace with peer institutions with well-developed online programs.

## Techno-Pedagogical Training

With the exception of undergraduate colleges that emphasize teaching, faculty in graduate schools are often rewarded more for research skills than teaching acumen. Most instructors learn to teach by doing, copying the kind of instruction they received in college and as teaching assistants. An assistant professor may have only practiced teaching a handful of courses before having to manage a 4–6 course teaching load. For instructors with limited to no experience teaching online, the abrupt shift to remote teaching proved highly disruptive and stressful, forcing many to do the best they could in a very difficult situation.

Delivering a course online is not simply recording and making available hour long lectures. Designing an effective online course requires partitioning didactic material into shorter “chunks” and micro-lectures followed by a variety of assessments such as quizzes, games, real-world based simulations, discussions led by students as moderators, icebreaker activities, and microblogging to keep students engaged (5). Online courses are designed with navigation and structured modules to facilitate how the student flows through the course. Modules should contain different types of assessments and activities to reinforce skills. These may be group-based or individual projects with appropriate selection of software and hardware (6). Instructors should sustain instructor presence and facilitate learner-to-learner interaction on a regular basis. Especially for online courses taught for the first time, formative evaluation is important to check how students are responding to the structure, navigation, activities, and peer interaction. These are some of the skills necessary to successfully develop and manage an online course (7).

Properly designed online courses require instructional design and technology expertise. Instructional designers have expertise in reworking instructional material for different modalities and instructional goals. It is also very useful to identify a group of instructors and colleagues experienced in online learning in order to share tips and advice. This could lead to learning communities within the organization to support each other as online courses are introduced and refined every semester. Schools and programs should ensure that instructors of all ranks have access to expertise and centers of teaching and learning. Centers of teaching and learning typically provide training in basic instructional design, accessibility and universal design for learning (UDL) principles, and adult pedagogy techniques.

Online modes offer opportunities that in-person courses do not: easier and more robust peer interaction and learning; access to more geographically diverse student bodies; different student demographics including older students, students with jobs and family obligations, students with physical disabilities; more time for students to interact with and learn from people within their communities; and greater and more even participation in course activities.

Many instructors have looked to technology as the solution, but technology only works if tailored to meet instructional goals that are grounded in effective and evidence-based pedagogy. Delivering online courses synchronously without modifying instructional strategy may lead to disappointing results and may exacerbate inequities, particularly among students from disadvantaged backgrounds (8). Just as sitting in a classroom does not ensure that a student is learning, being on a Zoom conference does not ensure learning; the mode of interaction in both cases needs to be designed to engage students in active and meaningful learning. Large variability in teaching quality already exists in traditional in-person classrooms. Online education may be particularly risky business for schools that do not have properly trained and supported instructors who can teach effectively online.

Regardless of modality, teaching should be a highly social process. It can be done effectively fully online with good design and appropriate technology. However, in order to achieve this, we need renewed thinking and investment in online education, training and other educational innovations. This will require coordination and commitment of senior leadership within learning institutions everywhere and willingness on the part of faculty to learn from this emergency and prepare for new modalities, expand the toolbox of teaching tools, and evolve as educators.

## Improving Student Readiness and Preparation for Online Education

Schools and programs should regularly collect data on student readiness for online learning. Schools should survey students to discover who needs equipment, services, and materials and follow up with an action plan to address these needs. Online courses typically require or propel students to develop good time management and self-organization skills. Designed to show students how to self-manage and organize, virtualized student orientations can help solidify these skills. Such orientations could include training on the learning management system, digital portfolio or online website building, training on accessing library services and career resources, and career-oriented skills-building. Following the training, learning should be tracked and assessed to ensure that all students achieve expected competencies in all relevant areas.

For students, interaction with peers and instructors both in and out of the classroom is an important part of the academic experience. Students will benefit from group-based collaboration in online courses and will use a variety of digital tools and spaces, increasingly important as the world moves toward more cloud-based collaboration across time zones, remote working models, and other technological advances. Online courses can provide an excellent opportunity to emphasize important skills such as time and project management, effective communication, and accountability. Properly designed online courses could complement in-person activities in blending learning formats leading to better learning outcomes than fully online or in-person alone (9). According to a recent Harvard Business Review article, “emotion” and “context” underpin critical “soft” skills that employers consider important when hiring: creative thinking, problem solving, effective communication, adaptive learning and good judgment. Technology, alone, through algorithms and artificial intelligence has not been able to reliably “emulate” these fundamentally human skills (10). As schools and programs, the use of collaboration and communication tools in school administration, research, and public programming can benefit from online education. Schools of public health could create a culture of hybrid digital and in-person collaboration that normalizes online education as well as teleworking, telehealth, and beyond.

## Reimagining Educational Offerings

Moving programs and courses online presents opportunities for learning institutions to evolve and grow. To keep pace with institutions that have successfully implemented online learning, schools and programs should create flexible and modular options in academic offerings. Coupled with the rise in cost of education and decreased perceived value in college education, consideration should be given to creating short certificate programs that focus on skills in demand that are not typically covered in current offerings. Targeted certificates and shorter credentialing options should be tied to meet labor demands and to help people upskill and find jobs. Examples include skills as diverse as change management, strategic communication, strategic planning, forecasting, social media marketing, media training, and conflict resolution (11). Online courses, especially asynchronous courses, may be convenient for busy working professionals from different sectors. The application of public health concepts and skills toward different professions adds value and relevance to them.

The pandemic has accelerated renewed attention to online learning. It also exposed how vulnerable society is to threats to health and well-being. Schools, programs, and educational institutions all around the globe must contend with new realities that are not likely to disappear in the future. As part of its long-term strategy, public health programs should look further afield to different disciplines, including business, engineering, art and design and others, to determine what content and skills can and should be taught.

The industry that develops educational technology for learning and teaching is growing rapidly and is poorly regulated (12). Some education technology investors have bold and ambitious visions including bypassing universities and colleges altogether. Some are working closely with employers to match students with employment via alternative credentialing (13). As a profession, we should not divert attention from these developments but must seriously contend with them. Public health should meet the new challenges ahead by supporting and training instructors to perform well with new and mixed modalities, by supporting students with new skills and competencies, and by positioning its schools and programs to become more adaptive in the face of emergencies.

## Author Contributions

SC wrote the initial draft. NC and IK made major edits. TH edited the paper in the final stage. All authors contributed to the article and approved the submitted version.

## Conflict of Interest

The authors declare that the research was conducted in the absence of any commercial or financial relationships that could be construed as a potential conflict of interest.

## Publisher's Note

All claims expressed in this article are solely those of the authors and do not necessarily represent those of their affiliated organizations, or those of the publisher, the editors and the reviewers. Any product that may be evaluated in this article, or claim that may be made by its manufacturer, is not guaranteed or endorsed by the publisher.
